# A Patient With Multiple Carbapenemase Producers Including an Unusual *Citrobacter sedlakii* Hosting an IncC *bla*_NDM-1_- and *armA*-carrying Plasmid

**DOI:** 10.20411/pai.v6i2.482

**Published:** 2021-11-22

**Authors:** Aline I. Moser, Peter M. Keller, Edgar I. Campos-Madueno, Laurent Poirel, Patrice Nordmann, Andrea Endimiani

**Affiliations:** 1 Institute for Infectious Diseases (IFIK), University of Bern, Bern, Switzerland; 2 Graduate School of Cellular and Biomedical Sciences, University of Bern, Bern, Switzerland; 3 Emerging Antibiotic Resistance Unit, Medical and Molecular Microbiology, Department of Medicine, University of Fribourg, Fribourg, Switzerland; 4 French INSERM European Unit, University of Fribourg (LEA-IAME), Fribourg, Switzerland; 5 National Reference Center for Emerging Antibiotic Resistance (NARA), Fribourg, Switzerland

**Keywords:** carbapenemases, NDM-1, Enterobacterales, ArmA, plasmid, CPE

## Abstract

**Background.:**

Patients colonized with multiple species of carbapenemase-producing Enterobacterales (CPE) are increasingly observed. This phenomenon can be due to the high local prevalence of these pathogens, the presence of important host risk factors, and the great genetic promiscuity of some carbapenemase genes.

**Methods.:**

We analyzed 4 CPE (*Escherichia coli, Klebsiella pneumoniae, Providencia stuartii, Citrobacter sedlakii*), 1 extended-spectrum cephalosporin-resistant *K. pneumoniae* (ESC-R-*Kp*), and 1 carbapenemase-producing *Acinetobacter baumannii* simultaneously isolated from a patient transferred from Macedonia. Susceptibility tests were performed using a microdilution MIC system. The complete genome sequences were obtained by using both short-read and long-read whole-genome sequencing technologies.

**Results.:**

All CPE presented high-level resistance to all aminoglycosides due to the expression of the *armA* 16S rRNA methylase. In *C. sedlakii* and *E. coli* (ST69), both the carbapenemase *bla*_NDM-1_ and *armA* genes were located on an identical IncC plasmid of type 1a. The *K. pneumoniae* (ST268) and *P. stuartii* carried chromosomal *bla*_NDM-1_ and *bla*_OXA-48_, respectively, while the ESC-R-*Kp* (ST395) harbored a plasmid-located *bla*_CTX-M-15_. In the latter 3 isolates, *armA*-harboring IncC plasmids similar to plasmids found in *C. sedlakii* and *E. coli* were also detected. The *A. baumannii* strain possessed the *bla*_OXA-40_ carbapenemase gene.

**Conclusions.:**

The characterization of the genetic organization of IncC-type plasmids harbored by 3 different species from the same patient offered insights into the evolution of these broad-host-range plasmids. Moreover, we characterized here the first complete genome sequence of a carbapenemase-producing *C. sedlakii* strain, providing a reference for future studies on this rarely reported species.

## INTRODUCTION

The spread of carbapenemase-producing Enterobacterales (CPE) represents a major public health issue. To date, KPC-2/-3- and OXA-48-producing *Escherichia coli* and *Klebsiella pneumoniae* isolates have been reported worldwide and in some geographic areas their prevalence is alarming [1, 2]. In addition, though less predominant, the NDM-producing species are of particular clinical concern because the NDM carbapenemase activity cannot be inhibited by clinically available β-lactamase inhibitors [3]. Moreover, the *bla*_NDM_ genes show great promiscuity since they can be located in different genetic environments, being either integrated into the chromosome or on extra-chromosomal mobile genetic elements (MGEs) among different bacterial species. In particular, some conjugative plasmids harbor additional antimicrobial resistance genes (ARGs) conferring co-resistances to other antibiotic families, such as the ArmA 16S rRNA methylase enzyme that modifies the target of aminoglycosides resulting in resistance to all clinically-used aminoglycosides, including the most recently developed plazomicin [4–6].

In this overall scenario, reports of patients simultaneously infected and/or colonized with multiple species of CPE are becoming a source of real concern. Several cases of interspecies exchange of identical *bla*_KPC_- [7, 8], *bla*_OXA-48_- [9, 10], and *bla*_NDM-1_-carrying plasmids have been described [9, 11–13]. In particular, those involving the *bla*_NDM-1_ were mainly due to the horizontal spread of broad-host-range IncC plasmids (formerly IncA/C2) [14]. In such cases, 2 to 4 different CPE were isolated from the same subjects: *E. coli* and *K. pneumoniae* were usually involved in this phenomenon, but *Klebsiella oxytoca, Citrobacter freundii, Proteus mirabilis,* or *Morganella morganii* strains could also be encountered [9, 11, 13].

In this work, we report a clinical case of a patient being simultaneously colonized by 3 *bla*_NDM-1_- and one *bla*_OXA-48_-positive Enterobacterales, along with an *Acinetobacter baumannii* strain possessing a *bla*_OXA-40_ carbapenemase gene. Enterobacterales were characterized at the genomic level by implementing both short-read and long-read whole-genome sequencing (WGS) technologies. Above all, we provide here the first genomic characterization of a unique *bla*_NDM-1_- and *armA*-positive *Citrobacter sedlakii* isolate.

## MATERIALS AND METHODS

### Clinical case.

In December 2020, a Swiss man in his 20s was admitted at the Inselspital (Bern, Switzerland). The subject was transferred from Macedonia, where he had been hospitalized as a polytraumatized individual for 2 months (further detailed clinical data regarding this hospitalization are not available). For surveillance purpose, a rectal swab to screen for the presence of multidrug-resistant Gram-negative bacteria was withdrawn. Moreover, blood cultures, swabs from skin ulcers, and cultures from vascular catheters were also performed over the course of 14 days (Table 1). The patient was kept in isolation during these 2 weeks before transfer to another Swiss hospital. The present anonymized case description has been carried out in accordance with the Declaration of Helsinki. The patient has also signed a general consent.

**Table 1.** Summary of the samples and bacteria isolated from the patient during the routine tests

Day^a^	Sample taken and results (if any)^b^				
1	Three blood cultures: Negative *Corynebacterium* spp. *M. morganii*	Rectal swab: ***E. coli* 3347558 (CP)**^c^	Indwelling catheter tip: Negative	Nasal swab for MRSA: Negative	Wound swab (lower leg left): ***K. pneumoniae* (CP)** ***C. freundii* complex (CP)** ***A. baumannii* (CP)** *K. pneumoniae*^d^
	Swab at the insertion site of the venous catheter: ***A. baumannii* 3347684 I (CP)** *K. pneumoniae* 3347684 II^c^^, ^^d^	Swab of a sacral ulcer: ***P. stuartii* 3347685 (CP)**^c^ ***A. baumannii* (CP)** *K. pneumoniae*^d^	Swab at the insertion site of a permanent catheter: ***K. pneumoniae* (CP)** ***C. freundii* complex (CP)** ***A. baumannii* (CP)**	Swab of the tracheostomy tube wound: Negative	Wound swab (heel left): ***K. pneumoniae* (CP)** ***C. freundii* complex (CP)**
	Swab of the left external malleolus: ***K. pneumoniae* 3347689 I (CP**^c^ ***C. freundii* complex 3347689 II (CP)**^c^	Swab of the right external malleolus: Negative			
2	Two blood cultures: *Corynebacterium* spp. Negative	Catheter tip: *P. aeruginosa* ***A. baumannii* (CP)**	Urine (from permanent catheter): Negative	Wound biopsy (decubitus): Negative	Bone biopsy (sacrum): *M. morganii*
	Wound biopsy (malleolus): *P. aeruginosa*	Biopsy soft tissue (decubitus): *M. morganii*	Biopsy soft tissue (decubitus): *M. morganii*		
6	Two blood cultures: Both negatives				
12	Blood culture: *Candida albicans*	Wound swab (sacrum): *P. aeruginosa*			
13	Two blood cultures: Both had *Candida albicans*	Central venous catheter (jugular): *K. pneumoniae*^d^ *C. albicans*	Arterial catheter (femoral): Negative		
14^e^	Tracheo-bronchial fluid: *P. aeruginosa* ***A. baumannii* (CP)** *K. pneumoniae*^d^				

a. Days from the hospitalization (admission at our institution in Bern, Switzerland)

b. Gram-negatives non-susceptible to carbapenems are reported in bold. “CP” indicates that these strains were carbapenemase producers according to the results of the Rapid Carba NP, CarbAcineto NP, NG-Test CARBA-5 and/or eazyplex assays implemented by the routine laboratory.

c. These bacteria were selected for WGS. We show their MIC values and genetic data in Table 2 and Table 3, respectively.

d. This strain was extended-spectrum cephalosporin-resistant (ESC-R), but carbapenem susceptible (see Table 2)

e. The patient was transferred to another Swiss institution

### Species identification (ID) and antimicrobial susceptibility tests (ASTs).

ID was routinely obtained using the matrix assisted laser desorption/ionization time of flight mass spectrometry (MALDI-TOF-MS; Bruker); it was then achieved using WGS data and the implementation of the Type (Strain) Genome Server (https://tygs.dsmz.de/). ASTs were performed using the broth microdilution ESB1F and GNX2F Sensititre panels (Thermo Scientific). Minimum inhibitory concentrations (MICs) for antibiotics were interpreted according to the European Committee on Antimicrobial Susceptibility Testing (EUCAST) criteria (version 9.0, 2019).

### Detection of ESBL and carbapenemase-producing (CP) strains.

The Rapid ESBL NP, Carba NP, and CarbAcineto NP colorimetric tests, along with the NG-Test CARBA-5 and the eazyplex Superbug complete B assays, were used to screen strains showing reduced susceptibility to extended-spectrum cephalosporins (ESCs) and/or carbapenems [1, 15]. Enterobacterales were further characterized by implementing the WGS (see below), whereas the *A. baumannii* was typed with a PCR/sequencing approach [16].

### Whole-genome sequencing (WGS).

Both NovaSeq 6000 (NEBNext Ultra II DNA library prep kit for Illumina; 2 x 150-bp paired-end reads) and MinION (SQK-RBK004 library; FLO-MIN 106D R9 flow-cell; Oxford Nanopore) technologies were implemented to perform WGS as previously described with an average sequencing coverage of 190x [17, 18]. In short, sequencing adapters from both Illumina and Nanopore reads were removed using Trimmomatic (v0.36) and Porechop (v0.2.4), respectively. The hybrid assembly was generated using Unicycler (v0.4.8) with default settings. Annotation was performed with the NCBI pipeline, but insertion sequences (ISs) were manually curated with ISfinder (https://isfinder.biotoul.fr/). The final genome was analyzed using the overall tools of the Center for Genomic Epidemiology (www.genomicepidemiology.org/). Integrons were classified according to INTEGRALL (http://integrall.bio.ua.pt/). The average nucleotide identity (ANI) was calculated using the OrthoANIu Calculator (http://www.ezbiocloud.net/tools/ani).

The complete genome assemblies of the 5 Enterobacterales have been deposited in GenBank (CP071068-CP071089) under BioProject PRJNA698767.

## RESULTS AND DISCUSSION

### Samples and bacteria.

Numerous samples taken at the admission of the patient gave positive results for CPE (*E. coli, K. pneumoniae, C. freundii* complex, and *Providencia stuartii*) and for a CP *A. baumannii*. An ESC-resistant *K. pneumoniae* (ESC-R-*Kp*) and a carbapenem-resistant *P. aeruginosa* were also isolated in multiple specimens (Table 1).

To study the features of these MDR-Gram-negatives, 4 representative strains of the CPE species, the ESC-R-*Kp*, and the CP *A. baumannii* were selected for further phenotypic and molecular analyses. The antibiotic MICs for these 6 illustrative strains are depicted in Table 2. As expected, the 5 CP strains showed reduced susceptibility to carbapenems, but the 4 CPE also presented high-level resistance to all tested aminoglycosides. Moreover, the ESC-R-*Kp* showed a phenotype consistent with the extended-spectrum β-lactamase (ESBL) production.

**Table 2.** Phenotypic characterization of the 6 Gram-negatives isolated from the same patient

Antibiotics	Strain (species and lab code), sample and MICs (mg/L)^a^											
	*E. coli*^b^ 3347558 Rectal swab		*K. pneumoniae*^b^ 3347684 II Swab of the insertion site of venous catheter		*K. pneumoniae*^b^ 3347689 I Swab of the left external malleolus		*P. stuartii*^b^ 3347685 Swab of the sacral ulcer		*C. sedlakii*^b^ 3347689 II Swab of the left external malleolus		*A. baumannii* 3347684 I Swab of the insertion site of venous catheter	
Piperacillin-tazobactam	>64	R	<4	S	>64	R	>64	R	>64	R	>64	na
Ticarcillin-clavulanate	>128	R	128	R	>128	R	>128	R	>128	R	>128	na
Cefpodoxime	>32	R	>32	R	>32	R	>32	R	>32	R	nt	
Ceftazidime	>128	R	32	R	>128	R	128	R	>128	R	>128	na
Ceftazidime-clavulanate	>128	na	0.25	na	>128	na	128	na	>128	na	nt	
Ceftriaxone	>128	R	128	R	>128	R	>128	R	>128	R	nt	
Cefotaxime	>64	R	64	R	>64	R	>64	R	>64	R	>64	na
Cefotaxime-clavulanate	>64	na	<0.125	na	>64	na	>64	na	>64	na	nt	na
Cefepime	>16	R	8	R	>16	R	>16	R	>16	R	16	na
Aztreonam	>16	R	>16	R	>16	R	>16	R	>16	R	>16	na
Imipenem	1	S	<0.5	S	8	R	2	I	8	R	>8	R
Meropenem	<1	S	<1	S	>8	R	2	S	8	R	>8	R
Doripenem	0.5	na	<0.125	na	>2	na	1	na	>2	na	>2	na
Ertapenem	2	R	<0.25	S	>4	R	0.5	S	>4	R	>4	na
Gentamicin	>8	R	>16	R	>16	R	>8	R	>8	R	>8	R
Tobramycin	>8	R	>8	R	>8	R	>8	R	>8	R	4	S
Amikacin	>32	R	<4	S	>32	R	>32	R	>32	R	>32	R
Ciprofloxacin	1	R	2	R	>2	R	>2	R	<0.25	S	>2	R
Levofloxacin	<1	S	<1	S	8	R	>8	R	<1	S	4	R
Doxycycline	>16	na	<2	na	16	na	>16	na	<2	na	<2	na
Minocycline	8	na	<2	na	4	na	>16	na	<2	na	<2	na
Tigecycline	<0.25	S	0.5	na	1	na	2	na	0.5	S	<0.25	na
Trimethoprim/sulfamethoxazole	>4	R	2	S	>4	R	4	I	>4	R	<0.5	S
Colistin	<0.25	S	<0.25	S	<0.25	S	>4	na	<0.25	S	<0.25	S
Polymyxin B	0.5	na	0.5	na	0.5	na	>4	na	<0.25	na	<0.25	na

**Note.** R, resistant; I, susceptible, increased exposure; S, susceptible; na, not available or not applicable; nt, not tested

a MICs were obtained with microdilution Sensititre panel GNX2F and ESB1F and interpreted according to the EUCAST 2019 criteria (version 9.0). *A. baumannii* was tested using only the GNX2F panel.

b Species identification was obtained based on the WGS and implementing the hybrid WGS assembling

### Molecular features of the MDR bacteria.

As shown in Table 3, the gut flora of the patient was colonized with a sequence type (ST) 69 *E. coli* strain (named 3347558) possessing numerous ARGs, including the carbapenemase gene *bla*_NDM-1_, the ESBL gene *bla*_CTX-M-15_, and the 16S rRNA methylase gene *armA*. Of note, the pandemic ST69 lineage is rarely associated with *bla*_NDM-1_, and it has never been reported to contain simultaneously both *bla*_NDM-1_ and *armA* [19].

**Table 3.** Molecular characterization of the 5 Enterobacterales isolated from the same patient

Sequence ID	GenBank	Sequence type	Length (bp)	Inc group	Antimicrobial resistance genes (ARGs)^a^	Genetic environment of the main ARGs^a^
***E. coli,* 3347558, ST69** ^b^ ^, ^ ^c^						
3347558	CP071073	chromosome	5′631′396	-	mdf(A)	
p3347558_1	CP071074	plasmid	169′082	C type 1a	*dfrA14, arr-2, cmlA1, bla*_OXA-10_*, aadA1, sul1, aph(3*'*)-VI,* ***bla***_NDM-1,_ ***armA****, msr(E), mph(E), bla*_CMY-4_	IS*Aba125*-***bla***_NDM-1_-*ble* IS*Ec28*-***armA***-IS*Ec29*
p3347558_2	CP071075	plasmid	129′523	FIA, Y	*aph(3*"*)-Ib, aph(6)-Id, bla*_TEM-1B_*, mph(A), tet(B), dfrA14, sul2*	
p3347558_3	CP071076	plasmid	93′750	I1-I (Gamma)	*aac(3)-IId,* ***bla***_CTX-M-15_*, bla*_TEM-1B_	IS*26*-ΔIS*Ecp1*-***bla***_CTX-M-15_-*wbuC*-IS*26*
p3347558_4	CP071077	plasmid	64′917	FII	nd	
p3347558_5	CP071078	plasmid	5′167	nd	nd	
p3347558_6	CP071079	plasmid	4′072	nd	nd	
***K. pneumoniae,* 3347684 II, ST268** ^b^						
3347684 II	CP071080	chromosome	5′290′520	-	*bla*_SHV-11_ *, oqxB, fosA5*	
p3347684 II_1	CP071081	plasmid	155′851	FIB(K)	nd	
p3347684 II_2	CP071082	plasmid	110′998	FIB	nd	
p3347684 II_3	CP071083	plasmid	64′471	nd	*aac(3)-IIa, aac(6*'*)-Ib-cr, bla*_OXA-1_*,* ***bla***_CTX-M-15_*, qnrB1, catB3, dfrA14*	IS*Ecp1*-***bla***_CTX-M-15_-*wbuC*-Tn*3* family *tnp*
p3347684 II_4	CP071084	Plasmid	63′577	FII(Yp)	nd	
p3347684 II_5	CP071085	plasmid	4′251	Col(pHAD28)	nd	
***K. pneumoniae,* 3347689 I, ST395** ^b^						
3347689I	CP071086	chromosome	5′620′517	-	*aac(3)-IId, bla*_TEM-1B_*,* ***bla***_NDM-1_*, bla*_SHV-182_*, oqxA, oqxB, sul1, fosA*	IS*26*-ΔIS*Aba125*-***bla***_NDM-1_-*ble*-Δ*bla*_DHA-1_-*lysR-qacEΔ1*-IS*CR1*
p3347689I_1	CP071087	plasmid	222′786	C type 1a / R	*dfrA14, arr-2, cmlA1, bla*_OXA-10_*, aadA1, sul1, aph(3*'*)-VI,* ***armA****, msr(E), mph(E), bla*_CMY-4,_ *aac(3)-IIa, aac(6*'*)-Ib-cr, bla*_OXA-1_*,* ***bla***_CTX-M-15_*, tet(A), tet(R), catA1, dfrA1*	IS*Ec28*-***armA***-IS*Ec29* IS*26-*ORF*-wbuC*-***bla***_CTX-M-15_-IS*26*-Tn*3* family *tnp*
p3347689I_2	CP071088	plasmid	9′730	ColRNAI	Nd	
p3347689I_3	CP071089	plasmid	4′052	Col440II	Nd	
***P. stuartii,* 3347685** ^b^						
3347685	CP071068	chromosome	4′476′038	-	*aac(2*'*)-Ia, bla*_CTX-M-15_*,* ***bla***_OXA-48_*, tet(B), catA3, dfrA14*	IS*1999*-IS*1R*-***bla***_OXA-48_-*lysR*-IS*1999*
p3347685_1	CP071069	plasmid	188′750	C type 1b	*dfrA14, arr-2, cmlA1, bla*_OXA-10_*, aadA1, sul1,* ***armA****, msr(E), mph(E), bla*_CMY-4_, *aac(6*'*)Ib-cr, bla*_OXA-1_*, bla*_CTX-M-15_*, tet(A), aadA2, aph(3*"*)-Ib, aph(6)-Id, bla*_TEM-1B_*, dfrA12, sul2*	IS*Ec28*-***armA***-IS*Ec29*
***C. sedlakii*, 3347689 II** ^b^						
3347689 II	CP071070	chromosome	4′756′279	-	*bla*_SED-1_	
p3347689 II_1	CP071071	plasmid	166′860	C type 1a	*dfrA14, arr-2, cmlA1, bla*_OXA-10_*, aadA1, sul1,* ***bla***_NDM-1,_ ***armA****, msr(E), mph(E), bla*_CMY-4_	IS*Aba125*-***bla***_NDM-1_-*ble*_MBL_ IS*Ec28*-***armA***-IS*Ec29*
p3347689 II_2	CP071072	plasmid	44′080	R	*aadA1, dfrA1, aac(6*'*)-Ib-cr, bla*_OXA-1_*, catB3, sul1, mph(A), bla*_SHV-12_	

**Note.** nd, none detected

a The main ARGs are in bold

b All sequences were obtained by a hybrid WGS sequencing approach combining Illumina and Nanopore reads

c The upstream region of the chromosomal AmpC did not contain mutations able to improve the expression of the *bla* gene

**Figure 1.**
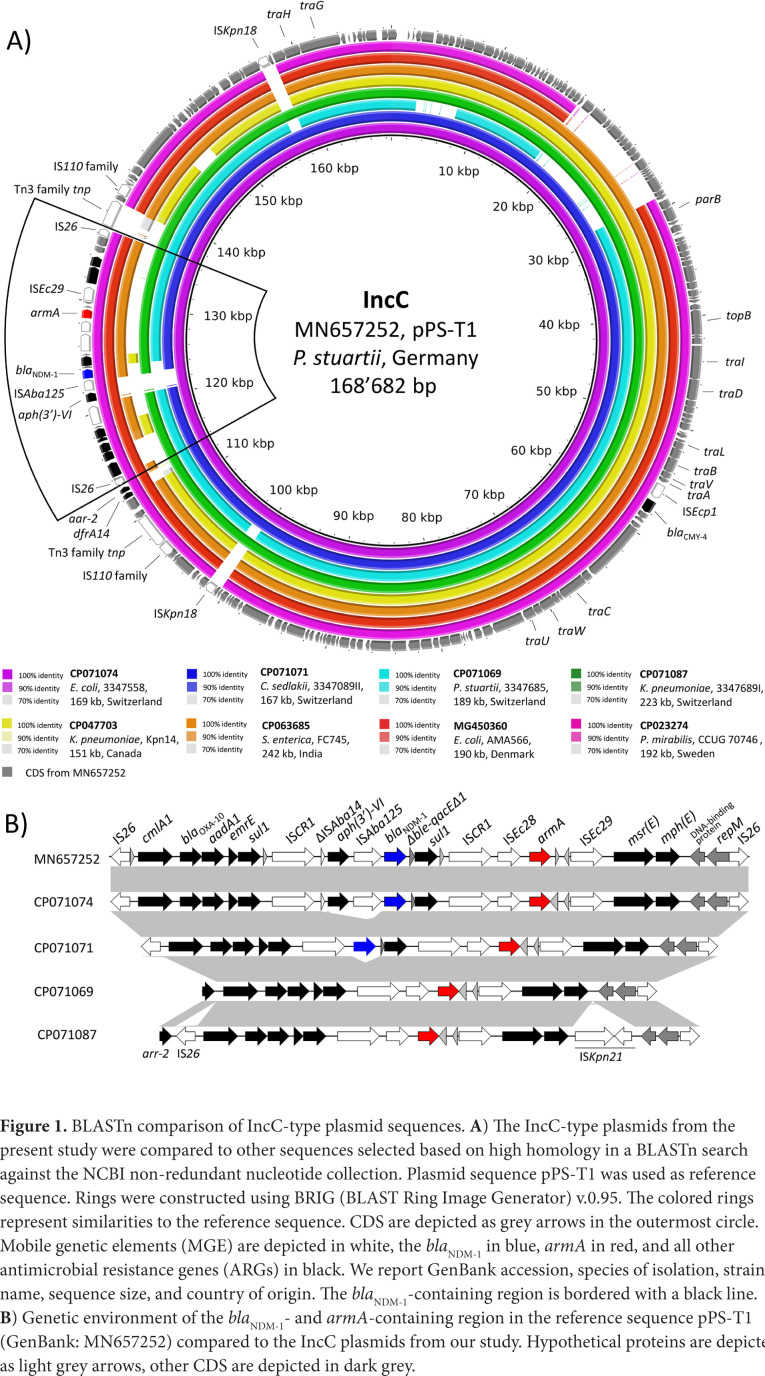
BLASTn comparison of IncC-type plasmid sequences. **A**) The IncC-type plasmids from the present study were compared to other sequences selected based on high homology in a BLASTn search against the NCBI non-redundant nucleotide collection. Plasmid sequence pPS-T1 was used as reference sequence. Rings were constructed using BRIG (BLAST Ring Image Generator) v.0.95. The colored rings represent similarities to the reference sequence. CDS are depicted as grey arrows in the outermost circle. Mobile genetic elements (MGE) are depicted in white, the *bla*_NDM-1_ in blue, *armA* in red, and all other antimicrobial resistance genes (ARGs) in black. We report GenBank accession, species of isolation, strain name, sequence size, and country of origin. The *bla*_NDM-1_-containing region is bordered with a black line. **B**) Genetic environment of the *bla*_NDM-1_- and *armA*-containing region in the reference sequence pPS-T1 (GenBank: MN657252) compared to the IncC plasmids from our study. Hypothetical proteins are depicted as light grey arrows, other CDS are depicted in dark grey.

In strain 3347558, *bla*_NDM-1_ and *armA* were co-carried on the ARI-A island of a multidrug resistance 169kb IncC plasmid of type 1a (p33477558_1) identical (identity, 99.81%) to pPS-T1 found in Germany (2015) in a *P. stuartii* strain of human origin (Figure 1A) [20]. In both p33477558_1 and pPS-T1, the *bla*_NDM-1_ was associated with IS*Aba125* and located between 2 IS*CR1* elements in a genetic environment identical to the one reported for the Serbian *P. aeruginosa* isolate MMA83 [4, 5, 21, 22]. Moreover, *armA* was positioned upstream of *bla*_NDM-1_, and it was organized in a classic element (IS*Ec28-armA*-IS*Ec29*) (Figure 1B) [23].

The patient also carried 2 *K. pneumoniae* strains belonging to different STs (Table 3).

The ESC-R-*Kp* strain 3347684 II was of ST268 and possessed a non-typeable plasmid of 64kb that carried the *bla*_CTX-M-15_. In contrast, the CP-*Kp* 3347689 I was of ST395 and possessed a chromosomally located *bla*_NDM-1_ along with a multidrug resistance 223kb multi-replicon IncC/R plasmid (p3347689I_1) carrying various ARGs including *armA* and *bla*_CTX-M-15_. The *bla*_NDM-1_ was located in a genetic context (IS*26*-ΔIS*Aba125-bla*_NDM-1_-*ble*-Δ*bla*_DHA-1_-*lysR-qacEΔ1*-IS*CR1*) identical (cover age, 100%; identity, 100%) to the one described for the IncL/M plasmid pNDM-OM from a clinical *K. pneumoniae* from Oman [24]. The *bla*_CTX-M-15_ was located on an IS*26*-flanked ~60kb IncR plasmid-derived sequence integrated into the ARI-A resistance island of the IncC plasmid. Except for the IncR plasmid-derived sequence, p3347689I_1 was highly similar (coverage, 98%; identity, 100%) to the IncC plasmid from *E. coli* 3347558 with *armA* located in an identical genetic context (Figure 1B). However, p3347689I_1 was missing the IS*CR1-aph(3*'*)-VI*-IS*Aba125-bla*_NDM-1_-Δ*ble- qacEΔ1-sul1* region, suggesting an acquisition event in p3347558_1 (Figure 1B). Finally, we note that ST395 is a globally successful lineage that usually carries *bla*_OXA-48_ and *bla*_KPC_ carbapenemase encoding-genes [25].

The CP *P. stuartii* strain 3347685 possessed *bla*_OXA-48_ and *armA*, consistent with the observed carbapenem and aminoglycoside phenotypic resistance (Table 2 and Table 3). We note that OXA-48-producing *P. stuartii* have been rarely reported and none of them co-produced ArmA or other 16S rRNA methylases leading to such pan-resistance to clinically used aminoglycosides.

In strain 3347685, *bla*_OXA-48_ was chromosome borne, and it was located within a Tn*1999.2* transposon [26]. The *armA* was located on a 189kb IncC plasmid of type 1b (p3347685_1) in a genetic context identical to the one in the type 1a plasmids p3347558_1 from *E. coli* and p3347689I_1 from the CP *K. pneumoniae* (Figure 1B). However, in contrast to the latter 2 plasmids, p3347685_1 was missing the type 1a patch region and carried the ARI-B resistance island in addition to ARI-A [14].

The CP *A. baumannii* isolate was resistant to all β-lactams including high-level resistance to carbapenems and carried the *bla*_OXA-40_ gene (Table 3). It was resistant to fluoroquinolones, but it remained susceptible to trimethoprim-sulfamethoxazole and to colistin (Table 2). It did not produce any additional ESBL or 16S rRNA methylase, remaining susceptible to tobramycin.

### Citrobacter sedlakii: genomic and plasmid characterizations.

Based on the WGS, the *C. freundii* complex strain 3347089 II was actually of *C. sedlakii* species. Moreover, the ANI values (≥98.98%) among our strain and the 6 *C. sedlakii* genome assemblies currently deposited in the NCBI genome database (accessed on 03/17/2021) confirmed the ID as *C. sedlakii* (data not shown). A core-genome analysis was performed including *C. sedlakii* 3347089 II, the 6 deposited genomes, and 3 assemblies deposited as *Citrobacter* spp. that were highly similar to 3347089 II based on the ANI values (>99%). As a result, no clonal relationship between the deposited assemblies and our *C. sedlakii* isolate could be observed (Supplemental Figure 1). It should be noted that only 2 *bla*_NDM_-positive *C. sedlakii* isolates were previously reported. However, the genomes of these 2 strains–respectively from Pakistan and Bangladesh–had not been sequenced [27, 28].

*C. sedlakii* 3347089 II carried a 167kb IncC type 1a plasmid and a 44kb IncR plasmid (Table 3). Remarkably, the IncC plasmid (p3347089II_1) was identical (identity, 100%) to the *bla*_NDM-1_ and *armA* carrying IncC plasmid from *E. coli* 3347558, except for the ΔIS*Aba14-aph(3*'*)-VI*-IS-*Aba125* region that was missing (Figure 1B). Comparison of the 3 IncC type 1a plasmids from our study suggested a common ancestor with a sequence similar to the one of p3347689I_1 from *K. pneumoniae*, but missing the IS*Kpn21* and the IncR plasmid-derived insertion sequence. The IS*CR1*-flanked *aph(3*'*)-VI, bla*_NDM-1_, and *sul1* and the *bla*_NDM-1_ and *sul1* in *E. coli* 3347558 and *C. sedlakii* 3347089 II, respectively, were likely acquired by a recombination event with the IS*CR1* element (Figure 1B) [29].

An analysis of the NCBI deposited *C. sedlakii* genomes revealed that the *Citrobacter* spp. strain 50677481 (GenBank: GCA_001463265; Supplemental Figure 1) possessed *bla*_NDM-1_ and *armA* to gether with an IncC plasmid. Therefore, we further analyzed its Illumina-derived WGS assembly. Mapping of the contigs to the plasmid sequence pPS-T1 from the German *P. stuartii* allowed the reconstruction of the complete plasmid sequence and showed that *Citrobacter* spp. 50677481 harbored an IncC plasmid identical to p3347558_1 from our *E. coli* isolate (coverage, 100%; identity, 99.98%) (data not shown). Remarkably, the *Citrobacter* spp. strain 50677481 was isolated in 2012 from a Norwegian patient with travel history to Serbia, suggesting a persistent and wide distribution of this multidrug resistance plasmid in the Balkan region [30].

Finally, the 44kb IncR plasmid (p3347089II_2) carried by our *C. sedlakii* contained multiple MGEs and ARGs including the integrons In*369* carrying *aadA1* and *dfrA1*, and In*1387* carrying *aac(6*'*)-Ib-cr, bla*_OXA-1_ and *catB3* (Table 3). The plasmid backbone of p3347089II_2 was identical (identity, 99.93%) to p12-6919.2, a 39kb IncR plasmid from a *Salmonella enterica* subsp. *enterica* isolate from Canada in 2012 (GenBank: CP039605).

## CONCLUSIONS

Although reports of CPE from Macedonia are missing, studies from neighboring countries reported concerning levels of NDM producers [31–33]. Therefore, the risk of importing CPE through the transfer of patients from these countries to those with a low prevalence is concerning. This phenomenon has been extensively discussed before (eg, in [34–36]). The present study underlined the importance of monitoring such cases to prevent the importation of multiple difficult-to-treat pathogens carrying novel antibiotic resistance traits.

Furthermore, we noted that while OXA-40-producing *A. baumannii* strains have been extensively described [1], the 4 CPE carried by the patient presented unusual patterns of antimicrobial resistance. In fact, all CPE were co-resistant to all aminoglycosides due to the production of the ArmA 16S rRNA methylase [6]. More importantly, *E. coli* 3347558 and *C. sedlakii* 3347089 II carried the *bla*_NDM-1_ and *armA* ARGs in an identical IncC type 1a plasmid suggesting an *in vivo* conjugation event. We also noted that this IncC type 1a plasmid was identical to one found in Germany in a *P. stuartii* isolate and to another one carried by a *Citrobacter* spp. strain linked to Serbia [20, 30].

Overall, our findings emphasize the potential of the IncC plasmids carrying life-threatening ARGs to spread worldwide among different Enterobacterales. The presence of these broad-host-range MGEs in rare enterobacterial species (eg, *C. sedlakii*) should be further investigated to better comprehend their origin and future evolution.

## SUPPLEMENTARY DATA

Supplementary materials are available at the Pathogens and Immunity website. Supplementary data may be provided by the authors to benefit the reader. Supplementary data are not copyedited and are the sole responsibility of the authors. Questions or comments related to supplementary materials should be addressed to the corresponding author.

## References

[1] Nordmann P, Poirel L. Epidemiology and Diagnostics of Carbapenem Resistance in Gram-negative Bacteria. *Clin Infect Dis.* 2019;69(Suppl 7):S521–S8. doi: 10.1093/cid/ciz824. PubMed PMID: 31724045; PMCID: PMC6853758. 31724045 PMC6853758

[2] Bonomo RA, Burd EM, Conly J, Limbago BM, Poirel L, Segre JA, Westblade LF. Carbapenemase-Producing Organisms: A Global Scourge. *Clin Infect Dis.* 2018;66(8):1290–7. doi: 10.1093/cid/cix893. PubMed PMID: 29165604; PMCID: PMC5884739. 29165604 PMC5884739

[3] Bush K. Past and Present Perspectives on beta-Lactamases. Antimicrob Agents *Chemother*. 2018;62(10). doi: 10.1128/AAC.01076-18. PubMed PMID: 30061284; PMCID: PMC6153792. PMC6153792 30061284

[4] Dortet L, Poirel L, Nordmann P. Worldwide dissemination of the NDM-type carbapenemases in Gram-negative bacteria. *Biomed Res Int.* 2014;2014:249856. doi: 10.1155/2014/249856. PubMed PMID: 24790993; PMCID: PMC3984790. 24790993 PMC3984790

[5] Wu W, Feng Y, Tang G, Qiao F, McNally A, Zong Z. NDM Metallo-beta-Lactamases and Their Bacterial Producers in Health Care Settings. *Clin Microbiol Rev.* 2019;32(2). doi: 10.1128/CMR.00115-18. PubMed PMID: 30700432; PMCID: PMC6431124. PMC6431124 30700432

[6] Wachino JI, Doi Y, Arakawa Y. Aminoglycoside Resistance: Updates with a Focus on Acquired 16S Ribosomal RNA Methyltransferases. *Infect Dis Clin North Am.* 2020;34(4):887–902. doi: 10.1016/j.idc.2020.06.002. PubMed PMID: 33011054. 33011054 PMC10927307

[7] Tijet N, Muller MP, Matukas LM, Khan A, Patel SN, Melano RG. Lateral dissemination and inter-patient transmission of blaKPC-3: role of a conjugative plasmid in spreading carbapenem resistance. *J Antimicrob Chemother*. 2016;71(2):344–7. doi: 10.1093/jac/dkv356. PubMed PMID: 26518052. 26518052

[8] Gona F, Barbera F, Pasquariello AC, Grossi P, Gridelli B, Mezzatesta ML, Caio C, Stefani S, Conaldi PG. In vivo multiclonal transfer of bla(KPC-3) from Klebsiella pneumoniae to Escherichia coli in surgery patients. *Clin Microbiol Infect.* 2014;20(10):O633–5. doi: 10.1111/1469-0691.12577. PubMed PMID: 24476498. 24476498

[9] Aires-de-Sousa M, Ortiz de la Rosa JM, Goncalves ML, Costa A, Nordmann P, Poirel L. Occurrence of NDM-1-producing Morganella morganii and Proteus mirabilis in a single patient in Portugal: probable in vivo transfer by conjugation. *J Antimicrob Chemother.* 2020;75(4):903–6. doi: 10.1093/jac/dkz542. PubMed PMID: 31971235. 31971235

[10] Arana DM, Saez D, Garcia-Hierro P, Bautista V, Fernandez-Romero S, Angel de la Cal M, Alos JI, Oteo J. Concurrent interspecies and clonal dissemination of OXA-48 carbapenemase. *Clin Microbiol Infect.* 2015;21(2):148 e1-4. doi: 10.1016/j.cmi.2014.07.008. PubMed PMID: 25596781. 25596781

[11] Hammerum AM, Hansen F, Nielsen HL, Jakobsen L, Stegger M, Andersen PS, Jensen P, Nielsen TK, Hansen LH, Hasman H, Fuglsang-Damgaard D. Use of WGS data for investigation of a long-term NDM-1-producing Citrobacter freundii outbreak and secondary in vivo spread of blaNDM-1 to Escherichia coli, Klebsiella pneumoniae and Klebsiella oxytoca. *J Antimicrob Chemother.* 2016;71(11):3117–24. doi: 10.1093/jac/dkw289. PubMed PMID: 27494919. 27494919

[12] Zhu B, Ying C, Xu H, Ying J. Coexistence of NDM-1-producing Escherichia coli and Citrobacter freundii in the same patient. *J Glob Antimicrob Resist.* 2018;15:79–81. doi: 10.1016/j.jgar.2018.04.013. PubMed PMID: 29727717. 29727717

[13] Bosch T, Lutgens SPM, Hermans MHA, Wever PC, Schneeberger PM, Renders NHM, Leenders A, Kluytmans J, Schoffelen A, Notermans D, Witteveen S, Bathoorn E, Schouls LM. Outbreak of NDM-1-Producing Klebsiella pneumoniae in a Dutch Hospital, with Interspecies Transfer of the Resistance Plasmid and Unexpected Occurrence in Unrelated Health Care Centers. *J Clin Microbiol.* 2017;55(8):2380–90. doi: 10.1128/JCM.00535-17. PubMed PMID: 28515215; PMCID: PMC5527415. 28515215 PMC5527415

[14] Ambrose SJ, Harmer CJ, Hall RM. Evolution and typing of IncC plasmids contributing to antibiotic resistance in Gram-negative bacteria. *Plasmid*. 2018;99:40–55. doi: 10.1016/j.plasmid.2018.08.001. PubMed PMID: 30081066. 30081066

[15] Endimiani A, Ramette A, Rhoads DD, Jacobs MR. The Evolving Role of the Clinical Microbiology Laboratory in Identifying Resistance in Gram-Negative Bacteria: An Update. *Infect Dis Clin North Am*. 2020;34(4):659–76. doi: 10.1016/j.idc.2020.08.001. PubMed PMID: 33011047. 33011047

[16] Bonnin RA, Nordmann P, Poirel L. Screening and deciphering antibiotic resistance in Acinetobacter baumannii: a state of the art. *Expert Rev Anti Infect Ther.* 2013;11(6):571–83. doi: 10.1586/eri.13.38. PubMed PMID: 23750729. 23750729

[17] Campos-Madueno EI, Bernasconi OJ, Moser AI, Keller PM, Luzzaro F, Maffioli C, Bodmer T, Kronenberg A, Endimiani A. Rapid Increase of CTX-M-Producing Shigella sonnei Isolates in Switzerland Due to Spread of Common Plasmids and International Clones. *Antimicrob Agents Chemother.* 2020;64(10). doi: 10.1128/AAC.01057-20. PubMed PMID: 32718957; PMCID: PMC7508577. PMC7508577 32718957

[18] Campos-Madueno EI, Gmuer C, Risch M, Bodmer T, Endimiani A. Characterisation of a new blaVIM-1-carrying IncN2 plasmid from an Enterobacter hormaechei subsp. steigerwaltii. *J Glob Antimicrob Resist.* 2021;24:325–7. doi: 10.1016/j.jgar.2021.01.017. PubMed PMID: 33571706. 33571706

[19] Dadashi M, Yaslianifard S, Hajikhani B, Kabir K, Owlia P, Goudarzi M, Hakemivala M, Darban-Sarokhalil D. Frequency distribution, genotypes and prevalent sequence types of New Delhi metallo-beta-lactamase-producing Escherichia coli among clinical isolates around the world: A review. *J Glob Antimicrob Resist.* 2019;19:284–93. doi: 10.1016/j.jgar.2019.06.008. PubMed PMID: 31212107. 31212107

[20] Weber RE, Pietsch M, Fruhauf A, Pfeifer Y, Martin M, Luft D, Gatermann S, Pfennigwerth N, Kaase M, Werner G, Fuchs S. IS26-Mediated Transfer of bla NDM-1 as the Main Route of Resistance Transmission During a Polyclonal, Multispecies Outbreak in a German Hospital. *Front Microbiol.* 2019;10:2817. doi: 10.3389/fmicb.2019.02817. PubMed PMID: 31921015; PMCID: PMC6929489. 31921015 PMC6929489

[21] Jovcic B, Lepsanovic Z, Begovic J, Rakonjac B, Perovanovic J, Topisirovic L, Kojic M. The clinical isolate Pseudomonas aeruginosa MMA83 carries two copies of the blaNDM-1 gene in a novel genetic context. Antimicrob Agents *Chemother*. 2013;57(7):3405–7. doi: 10.1128/AAC.02312-12. PubMed PMID: 23612199; PMCID: PMC3697382. 23612199 PMC3697382

[22] Poirel L, Bonnin RA, Boulanger A, Schrenzel J, Kaase M, Nordmann P. Tn125-related acquisition of blaNDM-like genes in Acinetobacter baumannii. *Antimicrob Agents Chemother.* 2012;56(2):1087–9. doi: 10.1128/AAC.05620-11. PubMed PMID: 22143526; PMCID: PMC3264265. 22143526 PMC3264265

[23] Bercot B, Poirel L, Nordmann P. Plasmid-mediated 16S rRNA methylases among extended-spectrum beta-lactamase-producing Enterobacteriaceae isolates. *Antimicrob Agents Chemother.* 2008;52(12):4526–7. doi: 10.1128/AAC.00882-08. PubMed PMID: 18838598; PMCID: PMC2592896. 18838598 PMC2592896

[24] Bonnin RA, Nordmann P, Carattoli A, Poirel L. Comparative genomics of IncL/M-type plasmids: evolution by acquisition of resistance genes and insertion sequences. *Antimicrob Agents Chemother.* 2013;57(1):674–6. doi: 10.1128/AAC.01086-12. PubMed PMID: 23114767; PMCID: PMC3535931. 23114767 PMC3535931

[25] Di Pilato V, Errico G, Monaco M, Giani T, Del Grosso M, Antonelli A, David S, Lindh E, Camilli R, Aanensen DM, Rossolini GM, Pantosti A, pneumoniae A-ILSGoc-pK. The changing epidemiology of carbapenemase-producing Klebsiella pneumoniae in Italy: toward polyclonal evolution with emergence of high-risk lineages. *J Antimicrob Chemother.* 2021;76(2):355–61. doi: 10.1093/jac/dkaa431. PubMed PMID: 33188415. 33188415

[26] Pitout JDD, Peirano G, Kock MM, Strydom KA, Matsumura Y. The Global Ascendency of OXA-48-Type Carbapenemases. *Clin Microbiol Rev.* 2019;33(1). doi: 10.1128/CMR.00102-19. PubMed PMID: 31722889; PMCID: PMC6860007. PMC6860007 31722889

[27] Qamar MU, Walsh TR, Toleman MA, Tyrrell JM, Saleem S, Aboklaish A, Jahan S. Dissemination of genetically diverse NDM-1, -5, -7 producing-Gram-negative pathogens isolated from pediatric patients in Pakistan. *Future Microbiol.* 2019;14:691–704. doi: 10.2217/fmb-2019-0012. PubMed PMID: 31148474. 31148474

[28] Rakhi NN, Alam A, Sultana M, Rahaman MM, Hossain MA. Diversity of carbapenemases in clinical isolates: The emergence of blaVIM-5 in Bangladesh. *J Infect Chemother.* 2019;25(6):444–51. doi: 10.1016/j.jiac.2019.01.010. PubMed PMID: 30824303. 30824303

[29] Toleman MA, Bennett PM, Walsh TR. ISCR elements: novel gene-capturing systems of the 21st century? *Microbiol Mol Biol Rev.* 2006;70(2):296–316. doi: 10.1128/MMBR.00048-05. PubMed PMID: 16760305; PMCID: PMC1489542. 16760305 PMC1489542

[30] Samuelsen O, Overballe-Petersen S, Bjornholt JV, Brisse S, Doumith M, Woodford N, Hopkins KL, Aasnaes B, Haldorsen B, Sundsfjord A, Norwegian Study Group on CPE. Molecular and epidemiological characterization of carbapenemase-producing Enterobacteriaceae in Norway, 2007 to 2014. *PLoS One.* 2017;12(11):e0187832. doi: 10.1371/journal.pone.0187832. PubMed PMID: 29141051; PMCID: PMC5687771. 29141051 PMC5687771

[31] Savov E, Politi L, Spanakis N, Trifonova A, Kioseva E, Tsakris A. NDM-1 Hazard in the Balkan States: Evidence of the First Outbreak of NDM-1-Producing Klebsi-ella pneumoniae in Bulgaria. *Microb Drug Resist.* 2018;24(3):253–9. doi: 10.1089/mdr.2017.0230. PubMed PMID: 28876169. 28876169

[32] Brkic S, Bozic D, Stojanovic N, Vitorovic T, Topalov D, Jovanovic M, Stepanovic M, Cirkovic I. Antimicrobial Susceptibility and Molecular Characterization of Carbapenemase-Producing Enterobacter spp. Community Isolates in Belgrade, Serbia. *Microb Drug Resist.* 2020;26(4):378–84. doi: 10.1089/mdr.2019.0224. PubMed PMID: 31651210. 31651210

[33] Politi L, Gartzonika K, Spanakis N, Zarkotou O, Poulou A, Skoura L, Vrioni G, Tsakris A. Emergence of NDM-1-producing Klebsiella pneumoniae in Greece: evidence of a widespread clonal outbreak. *J Antimicrob Chemother.* 2019;74(8):2197–202. doi: 10.1093/jac/dkz176. PubMed PMID: 31065697. 31065697

[34] Seiffert SN, Perreten V, Johannes S, Droz S, Bodmer T, Endimiani A. OXA-48 carbapenemase-producing Salmonella enterica serovar Kentucky isolate of sequence type 198 in a patient transferred from Libya to Switzerland. *Antimicrob Agents Chemother.* 2014;58(4):2446–9. doi: 10.1128/AAC.02417-13. PubMed PMID: 24468781; PMCID: PMC4023741. 24468781 PMC4023741

[35] Seiffert SN, Marschall J, Perreten V, Carattoli A, Furrer H, Endimiani A. Emergence of Klebsiella pneumoniae co-producing NDM-1, OXA-48, CTX-M-15, CMY-16, QnrA and ArmA in Switzerland. *Int J Antimicrob Agents.* 2014;44(3):260–2. doi: 10.1016/j.ijantimicag.2014.05.008. PubMed PMID: 25123809. 25123809

[36] Clement M, Keller PM, Bernasconi OJ, Stirnimann G, Frey PM, Bloemberg GV, Sendi P, Endimiani A. First Clinical Case of In Vivo Acquisition of DHA-1 Plasmid-Mediated AmpC in a Salmonella enterica subsp. enterica Isolate. *Antimicrob Agents Chemother.* 2019;63(10). doi: 10.1128/AAC.00992-19. PubMed PMID: 31358582; PMCID: PMC6761535. PMC6761535 31358582

