# Supplementary material for: A Patient With Multiple Carbapenemase Producers Including an Unusual Citrobacter sedlakii Hosting an IncC blaNDM-1- and armA-carrying Plasmid
Source: Pathog Immun. 2021 Nov 22;6(2):119–34. doi: 10.20411/pai.v6i2.482 (PMC8714174; doi:10.20411/pai.v6i2.482)
Supplement: Supplemental Figure 1 [file pai-6-119-s01.pdf]

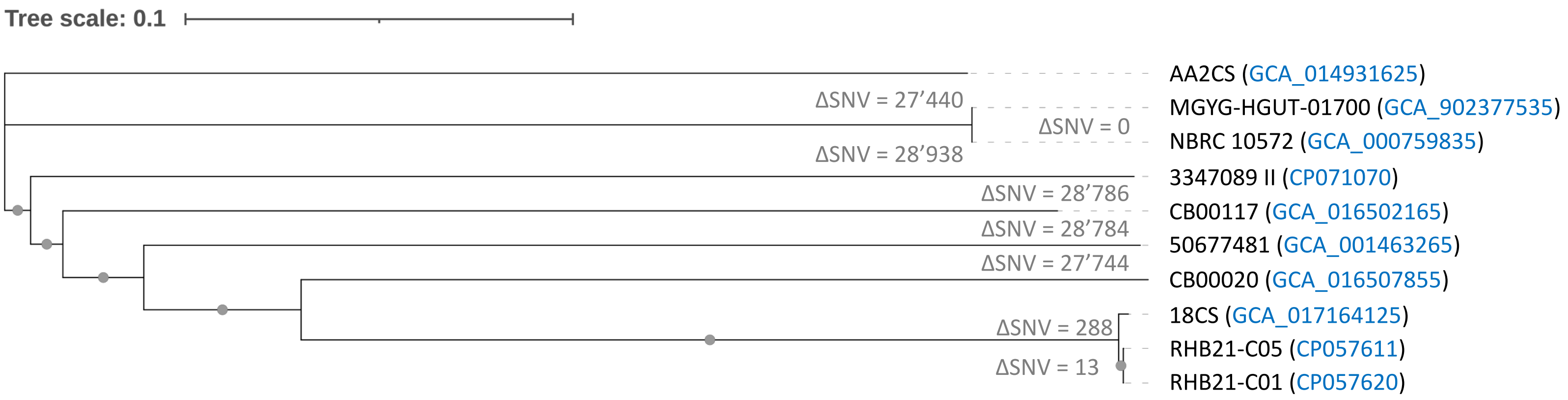

Figure S1. Core-genome comparison of *C. sedlakii* 3347089 II with all *C. sedlakii* genome assemblies deposited in the NCBI genome database and three assemblies deposited as *Citrobacter* spp. (strains 50677481, RHB21-C05, and RHB21-C01) (accessed on 03/17/2021). The assemblies are presented in a SNVs hierarchical clustering dendrogram tree obtained from the core-genome analysis performed using Parsnp (v1.2) and visualized with iTOL (https:/itol.embl.de/). The core-genome represents the total coverage (85.3%) among all sequences corresponding to 75’495 SNVs. The ΔSNV values indicate the number of non-identical SNVs between two strains. The grey circles on the branches indicate that the branch is supported by a bootstrap value >89. For each strain, the GenBank accession is reported in brackets.

Moser AI, Keller PM, Campos-Madueno EI, Poirel L, Nordmann P, Endimiani A. A patient with multiple carbapenemase producers including an unusual *Citrobacter sedlakii* hosting an IncC *bla*$_{NDM-1-}$ and *armA*-carrying plasmid. *Pathogens and Immunity*. 2021;6(2): 119-134. doi: 10.20411/pai.v6i2.482
